# Postictal Encephalopathy After Status Epilepticus: Outcome and Risk Factors

**DOI:** 10.1007/s12028-023-01868-1

**Published:** 2023-11-08

**Authors:** Clara Marie Bode, Simon Bruun Kristensen, Hanne Tanghus Olsen, Camilla Dyremose Cornwall, Lars Roberg, Olav Monsson, Thomas Krøigård, Palle Toft, Christoph P. Beier

**Affiliations:** 1 Department of Neurology, Odense University Hospital, Sdr. Boulevard 29, 5000 Odense, Denmark; 2 Department of Anesthesiology, Odense University Hospital, Odense, Denmark; 3 Department of Clinical Research, University of Southern Denmark, Odense, Denmark; 4 Department of Neurophysiology, Odense University Hospital, Odense, Denmark; 5 OPEN, Odense Patient Data Explorative Network, Odense University Hospital, Odense, Denmark

**Keywords:** Status epilepticus, Encephalopathy, Midazolam, Propofol, Outcome, Prognostication

## Abstract

**Background:**

Postictal encephalopathy is well known after status epilepticus (SE), but its prognostic impact and triggers are unknown. Here, we aimed to establish risk factors for the development of postictal encephalopathy and to study its impact on survival after discharge.

**Methods:**

This retrospective cohort study comprised adult patients diagnosed with first nonanoxic SE at Odense University Hospital between January 2008 and December 2017. Patients with ongoing SE at discharge or unknown treatment success were excluded. Postictal symptoms of encephalopathy were estimated retrospectively using the West Haven Criteria (WHC). WHC grade was determined for postictal day 1 to 14 or until the patient died or was discharged from the hospital. Cumulative postictal WHC during 14 days after SE-cessation was used to quantify postictal encephalopathy. Clinical characteristics, patient demographics, electroencephalographic and imaging features, and details on intensive care treatment were assessed from medical records.

**Results:**

Of all eligible patients (*n* = 232), 198 (85.3%) had at least WHC grade 2 postictal encephalopathy that lasted for > 14 days in 24.5% of the surviving patients. WHC grade at discharge was strongly associated with poor long-term survival (*p* < 0.001). Postictal encephalopathy was not associated with nonconvulsive SE, postictal changes on magnetic resonance imaging, or distinct ictal patterns on electroencephalography. Although duration of SE and treatment in the intensive care unit showed an association with cumulative postictal WHC grade, they were not independently associated with the degree of encephalopathy when controlling for confounders. In a linear regression model, etiology, duration of sedation, age, and premorbid modified Rankin Scale were significant and consistent predictors for higher cumulative postictal WHC grade. Exploratory analyses showed an association of a cumulative midazolam dosage (mg/kg/h) with higher cumulative postictal WHC grade.

**Discussion:**

In this cohort, postictal encephalopathy after SE was common and associated with poor long-term survival. Seizure characteristics were not independently associated with postictal encephalopathy; the underlying etiology, long (high-dose midazolam) sedation, high age, and poor premorbid condition were the major risk factors for its development.

**Supplementary Information:**

The online version contains supplementary material available at 10.1007/s12028-023-01868-1.

## Introduction

Status epilepticus (SE) is a serious neurological emergency characterized by abnormally prolonged seizures, which—after a period of time—can cause permanent neurological damage due to irreversible neuronal injury [1]. The overall mortality from SE is high and can reach up to 40% in refractory patients but varies depending on age, etiology, comorbidities, seizure duration, and level of consciousness [2–5]. Mortality of SE after discharge significantly exceeds in hospital mortality [6, 7], and attention regarding predicting of long-term mortality after SE has increased [8, 9]. However, the vast majority of studies primarily focused on the ictal rather than the postictal state.

The postictal state is considered a temporary state following the end of a seizure lasting minutes to several days, even weeks, in which the patient can develop sleepiness, confusion, altered level of consciousness, and focal neurological deficits, but also psychiatric or cognitive symptoms [10]. In a broader sense, these symptoms present a clinical picture of encephalopathy to a greater or lesser extent [11, 12]. In this context, encephalopathy can be defined as “widespread dysfunction or illness of the whole brain, essentially always affecting the neocortex broadly but often affecting subcortical structures as well” [12]. The clinical spectrum of encephalopathies may vary, but reduced alertness, confusion, and altered cognition are the key symptoms.

The pathophysiological mechanisms involved in the postictal state are not well understood, but changes in cerebral blood flow, neurotransmitter function [13], and side effects of medication are likely involved [14]. A previous study by Baumann et al. [15] found that postictal delirium is a frequent condition following SE treatment in the intensive care unit (ICU) and seen in more than half of the study population. Development of delirium was independently associated with alcohol and drug consumption [15]. However, postictal delirium is only one clinical manifestation that a patient may present following seizures, and the symptoms can be much more heterogenic [10]. In addition, there are no studies on the prognostic impact of postictal dysfunction on long-term survival after discharge.

Therefore, this study aimed to investigate the severity of the postictal encephalopathy in adult patients with first-time nonanoxic SE and its impact on long-term survival. In addition, potential risk factors associated with the emergence of postictal encephalopathy were analyzed.

## Materials and Methods

### Cohort

This retrospective cohort study was based on an already existing cohort that was previously used to study the associations of magnetic resonance imaging (MRI), electroencephalography (EEG), and new neurological deficits with long-term outcome [9, 16, 17]. In brief, all adult patients (≥ 18 years old) with first-time, nonanoxic SE (as defined in [1, 18]) who received a diagnosis and were treated at the University Hospital of Odense between January 2008 and December 2017 were retrospectively identified based on referrals for acute EEGs, from the International Classification of Diseases, Tenth Edition codes (DG40.3) at discharge and/or from clinical information by reviewing medical records. To further delineate the cohort relevant to this study examining the prognostic impact of postictal encephalopathy on long-term survival, patients who remained in SE at discharge were excluded. Further, patients without EEG and without unequivocal postictal clinical improvement after SE were excluded from analysis due to missing data (Supplementary Fig. 1). Patients with withdrawal of care during ongoing SE were excluded, whereas patients with withdrawal of care after successful treatment of SE remained in the study. The study complies with ethical standards defined by Danish legislation. Permission to handle medical records was acquired from the Danish Data Protection Agency (18/58576) and the Danish Health Authority (3-3013-2661/1). Data reporting followed the Strengthening the Reporting of Observational Studies in Epidemiology guideline [19].

### Evaluation of the Postictal State and Survival

For each patient, the postictal state was retrospectively estimated daily for 14 consecutive days following SE-cessation or until the patient returned to the habitual state and/or was discharged from the hospital. The time point of cessation of SE was identified using a combination of clinical and EEG-verified seizure freedom and was essentially based on the treating neurologists’ evaluation; new neurological deficits were determined by using the estimated National Institute of Health Stroke Scale score before admission and at discharge as described in [9].

The West Haven Criteria (WHC) for hepatic encephalopathy were used to quantify the severity of the clinical manifestations seen in the postictal state during the measured period [20, 21]. It comprises the following stages:

Grade 0: Asymptomatic patients.

Grade 1: Mild lack of awareness, euphoria or anxiety, shortened attention span, impaired performance of addition.

Grade 2: Lethargy or apathy, minimal disorientation for time or place, subtle personality changes, inappropriate behavior, impaired performance of subtraction.

Grade 3: Somnolence to semistupor but response to verbal stimuli, confusion, gross disorientation.

Grade 4: Coma.

Grade 5: Is not an official part the WHC but added as part of this study to account for the patients’ death when calculating the cumulative WHC grade after SE.

The evaluation was based on electronic medical records including all notes from nurses and therapist that were available for all patients. If a patient’s state varied during the day, the worst state was graded according to the WHC. All estimates of the WHC grade were performed by a single rater (C.M.B.) to avoid interrater bias. After the assessments were completed, the rater, anonymized to the results of the first assessment, evaluated the postictal state once again for the first 15 patients, which confirmed the initial results, and no changes of the initial assessment had to be made. The cumulative postictal WHC grade during the first 14 days was defined as the sum of the WHC grades from day 1 to 14. If a patient died before day 14, the patient received a score of 5 for the remaining days. If a patient was discharged before day 14, the patient was scored with a fictive score of 0 for the days after discharge. We defined a cumulative postictal WHC grade of 0–20 as “mild,” 21–40 as “moderate,” and values > 40 as “severe/prolonged” postictal encephalopathy.

In patients with persistent postictal impairment of consciousness, a 20–30-min spot EEG was routinely performed to exclude persistent nonconvulsive SE (NCSE).

Survival data were available for all patients due to the linkage of the electronic medical records and the Danish Central Person Register [6, 9, 16, 17].

### Clinical Characteristics

Clinical characteristics of the cohort were assessed as described previously [9] and were available for this study. SE was classified as described by Trinka et al. [1].The following parameters were assessed based on the patients’ electronic medical records: sex, age, weight, Charlson Comorbidity Index (CCI) score (without age criterion) [22], SE duration, modified Rankin Scale before admission, etiology as classified in [9], diagnosis of possible NCSE based on the Salzburg criteria [16], worst seizure type [23], and history of epilepsy [23].

Data on ictal MRI changes were previously published in [17]. In brief, postictal MRI changes were identified retrospectively in patients with SE and available standard 3T MRI (diffusion-weighted images) taken under SE or within 1 week after cessation of SE (C.D.C.). All available ictal EEGs were analyzed and classified retrospectively by L.E.R., O.M., and T.K. based on the Salzburg criteria [24]. Data were previously published in Monsson et al. [16].

Cumulative doses of midazolam and propofol were obtained retrospectively using the archive function within Cambio Clinical Information System (version 4.9.0.1) by S.B.K., H.T.O., and P.T. The cumulative doses were calculated from the start of the continuous infusion of a given sedative as part of SE treatment until stop of sedation (excluding opioids). If detailed information on infusion was not available, doses were estimated using the electronic medical records, administration lists, and the average rate of actual administered sedatives in the ICU.

### Statistics

Data were stored using RedCap (Vanderbilt University) [25]. Statistical analyses were performed using IBM SPSS 29. Percentages and frequencies, medians/means and interquartile ranges (IQRs) were given for descriptive analysis. Long-term survival was addressed using Kaplan–Meier estimator, and log-rank test was used to determine statistical significance. *p* values < 0.05 were considered significant without correction for multiple testing.

Univariable comparisons of sex, age group, etiology, diagnosis of possible NCSE, worst seizure type, history of epilepsy, and the primary outcome of these groups were performed with the *χ*^2^ for categorical variables and the Kruskal–Wallis test for numeric data.

Automatic linear modeling was used for the identification of major contributor of postictal encephalopathy. The cumulative WHC was used as continuous variable and end point; age, CCI, etiological groups, cumulative midazolam and propofol doses, modified Rankin Scale before admission, duration of SE, worst seizure type, and duration of sedation were putative predictors in the model. Variables were chosen based on assumed clinical relevance and low risk of collinearity. The identified significant predictors were then used as covariates in a linear regression model (method: enter) with cumulative postictal WHC as end point. The model did not account for heteroscedasticity. Significance of the model was assessed using *F*-statistic and goodness-of-fit was assessed with *R*-square. Effect sizes were measured with coefficients *β* and corresponding 95% confidence intervals.

## Results

### Patient Cohort

**Fig. 1:**
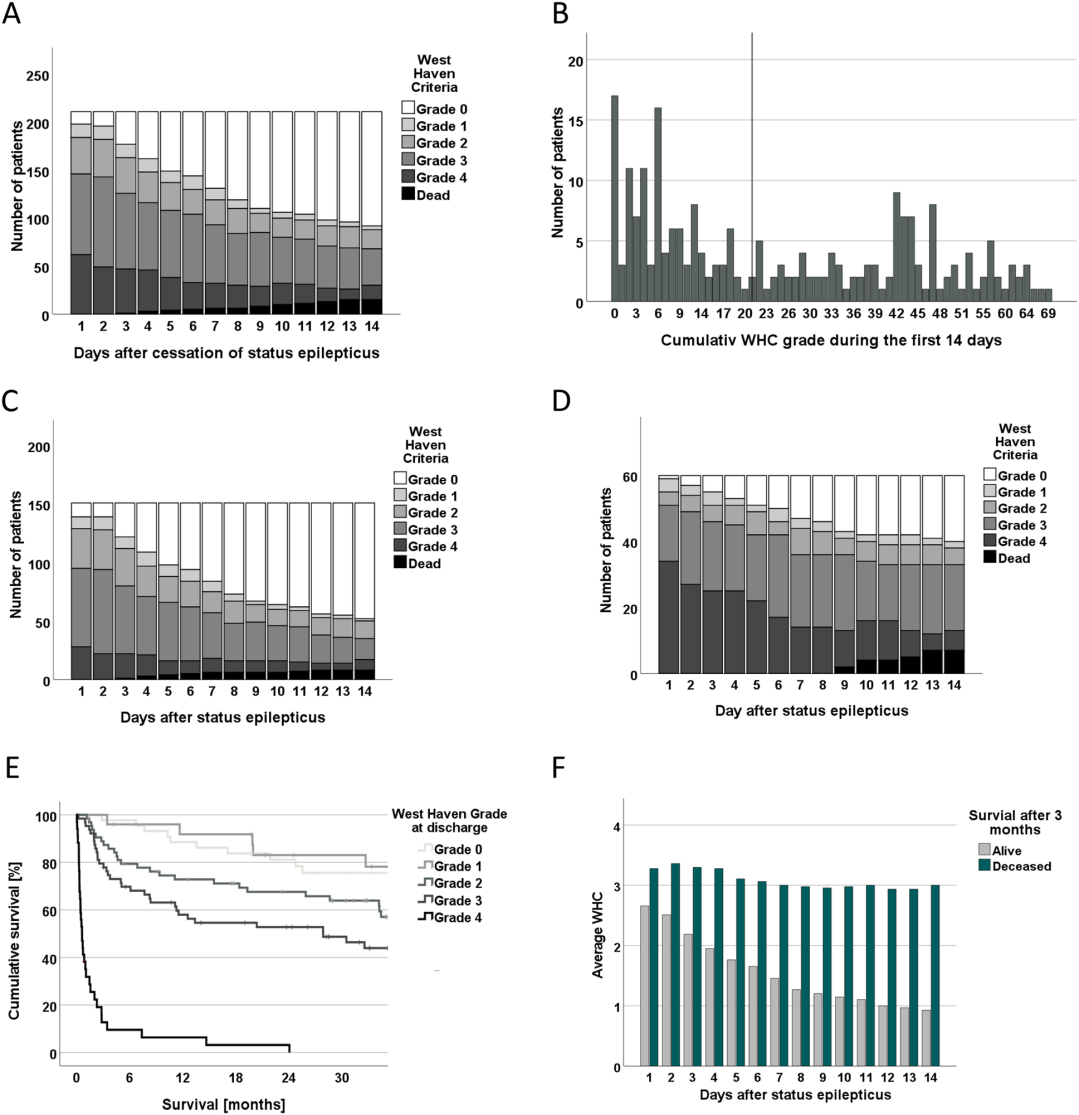
Postictal encephalopathy after status epilepticus (SE). **a** The WHC grades of all patients in the study during the first 14 days after cessation of SE are given. **b** Cumulative postictal WHC grades for all patients are given. The black vertical line gives the median. **c**, **d** The WHC grades of patients during the first 14 days after cessation of SE are given for (**c**) patient treated at the general ward for SE (*n* = 169) and for (**d**) patients treated in the ICU (*n* = 63). (**e**) Kaplan–Meier survival curves are shown for patients, depending on their WHC grade at discharge. Note that only patients discharged alive are included in this analysis. All survival curves apart from grade 0 and 1 and grade 2 and 3 differed significantly in pairwise log-rank test with a *p* value < 0.05. (**f**) Average postictal WHC grade depending on the survival status after 3 months. WHC, West Haven Criteria

Clinical and demographic data of the full cohort are shown in Table 1 and Supplementary Table 1. Supplementary Fig. 1 provides an overview of patient identification, screening, and selection that are compliant with the Strengthening the Reporting of Observational Studies in Epidemiology guidelines. Of all eligible patients (*n* = 232), 198 (85.3%) had WHC grade 2–4 postictal encephalopathy that persisted in 24.5% of all patients surviving for > 14 days after cessation of SE (Fig. 1a). Figure 1b gives an overview of the distribution of the cumulative postictal WHC grade of all eligible patients. The average cumulative postictal WHC grade during the first 14 days after seizure cessation was 24.6 (median 20.5; IQR 8–43).

**Table 1:** Patient demographics (*n* = 232)

Demographic		Count or mean	Percentage (%) or IQR
Cohort of patients with successful treatment of SE (n = 208)			
Sex	Female	118	50.9
	Male	114	49.1
Age	< 40	13	5.6
	40–70	97	41.8
	> 70	122	52.6
Etiology	Acute stroke	23	9.9
	Infectious	22	9.5
	Intoxication/metabolic	38	16.4
	Other acute symptomatic	11	4.7
	Remote symptomatic	74	31.9
	Progressive	35	15.1
	Cryptogenic	22	9.5
	Electroclin syndromes	7	3.0
Worst seizure type	Focal (un)aware/absence	80	34.5
	Tonic–clonic	89	38.4
	NCSE in coma	63	27.2
NCSE (Salzburg criteria)	Definite NCSE	124	58.8
	Possible NCSE	44	20.9
	No NCSE	87	41.2
WHC day 1	Grade 0	19	8.2
	Grade 1	15	6.5
	Grade 2	47	20.3
	Grade 3	88	37.9
	Grade 4	63	27.2
WHC discharge	Grade 0	44	19.0
	Grade 1	25	10.8
	Grade 2	64	27.6
	Grade 3	65	28.0
	Grade 4	34	14.7
Other characteristics	Modified Rankin scale (discharge)	3.2	2.0–4.0
	Charlson comorbidity index (without age)	4.3	2.0–6.0
Postictal MRI changes	Yes	19	82.1
	No	87	17.9
Patient not treated at ICU for SE (n = *154)*			
	Duration of SE (h)	102.6	15.4–134.7
	Time in hospital (d)	17.6	5.0–23.5
Patient treated at ICU for SE (n = *54)*			
	Propofol treatment	51	80.5
	Midazolam treatment	47	74.6
	Duration of SE (h)	145.9	28.4–181.5
	Duration of sedation (h)	58.9	5.0–67.0
	Number of sedation cycles	1.80	1.0–2.0
	Time in hospital (d)	31.9	9.0–40.0

*ICU* intensive care unit, *IQR* interquartile range, *MRI* magnetic resonance imaging, *NCSE* nonconvulsive status epilepticus, *SE* status epilepticus, *WHC* West Haven criteria

### Postictal Encephalopathy in the Patient Cohort

The temporal development of the patients’ postictal encephalopathy is illustrated in Fig. 1a for all patients, in Fig. 1c for patients who did not receive ICU treatment, and in Fig. 1d for patients treated in the ICU. The cumulative postictal WHC grade during the first 14 days after seizures cessation was not associated with the risk of new neurological deficits in patients with moderate-to-high cumulative postictal WHC grades (Supplementary Fig. 1a). Both the WHC grade at discharge (Fig. 1e) and the cumulative WHC (Supplementary Fig. 2) were significantly associated with survival. However, the prognostic impact of the cumulative WHC grade during the first 14 days was lower as compared with the estimated degree of encephalopathy at discharge. The association of the postictal WHC grade and survival became stronger with increasing duration of the encephalopathy (Fig. 1f).

### Risk Factors for Postictal Encephalopathy

**Fig. 2:**
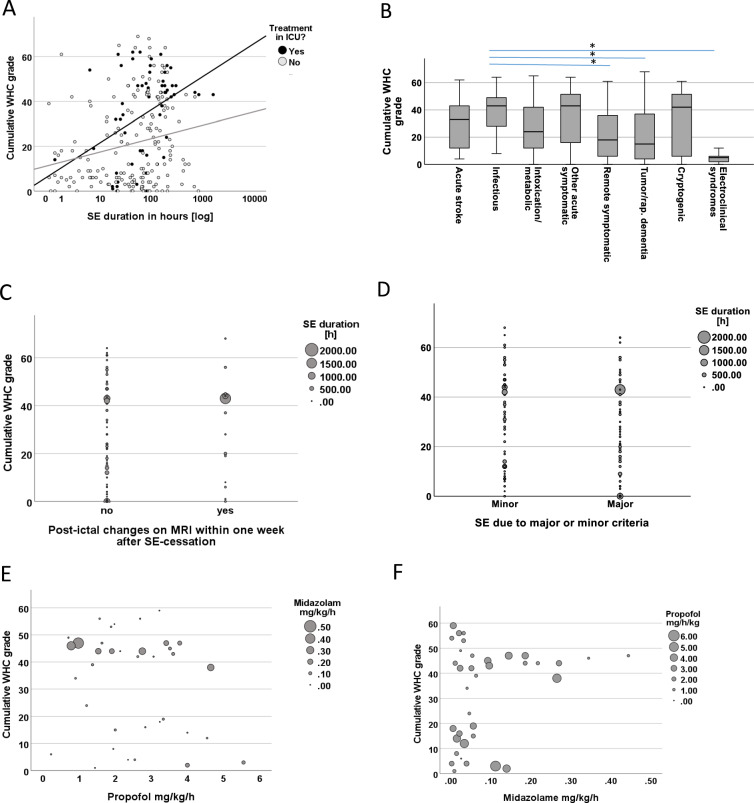
Risk factors for postictal encephalopathy. **a** The association of duration of SE, cumulative postictal WHC grade and treatment in the ICU are illustrated. **b** Association between etiology and cumulative postictal WHC grade is given. Asterisks indicate statistically significant differences (*p* < 0.05, Kruskal–Wallis test follow by control for multiple testing using the Bonferroni method). **c**, **d** The cumulative postictal WHC grade in patients with and without (**c**) ictal MRI changes and in patients with (**d**) nonconvulsive status epilepticus diagnosed using the minor or major criteria of the Salzburg criteria. Differences were not statistically significant between the groups. **e**, **f** Every dot in the figures represents data from an individual patient. **e** The association of the cumulative postictal WHC and the average hourly weight-corrected propofol doses are given. To illustrate the impact of midazolam treatment on postictal encephalopathy, the size of the dots gives the average hourly weight-corrected midazolam dose. **f** The same data as in (**e**) are given, however, the *x*-axis now gives the average hourly weight-corrected midazolam doses. To illustrate the impact of propofol treatment on postictal encephalopathy, the size of the dots gives the average hourly weight-corrected propofol dose. ICU, intensive care unit, MRI, magnetic resonance imaging, SE, status epilepticus, WHC, West Haven Criteria

Figure 2a illustrates the association of treatment in the ICU, duration of SE, and the cumulative postictal WHC grade. The cumulative postictal WHC grade depended on etiology. Figure 2b gives the cumulative WHC grade depending on the underlying etiology. It was highest in patients with infectious etiologies and lowest in patients with SE due to electro-clinical syndromes. Postictal MRI changes (Fig. 2c), NCSE (Table 2), and SE-defining electroencephalographic features (Fig. 2d) were not associated with the cumulative postictal WHC grade. Table 2 and Supplementary Table 2 gives univariate and multivariate associations of putative risks factors comparing patients with a cumulative postictal WHC grade above or below the median. Coma at admission, etiology, and duration of sedation showed independent associations. An automated regression model with cumulative postictal WHC grade as the end point and age, duration of SE, treatment in ICU, duration of sedation, NCSE, etiology, CCI, modified Rankin Scale before admission, average midazolam dose/day, average propofol dose/day, or type of antiseizure treatment (valproic acid, levetiracetam, fenytoin, lacosamide) as putative predictors identified etiology as most important risk factor, followed by duration of sedation, age, worst seizure type, and modified Rankin Scale before admission (data not shown). A linear regression model confirmed the results (Table 3); notably CCI reached significance in some but not all models. The independent association of etiology, duration of sedation, age, and modified Rankin Scale remained stable and consistent in various additional models established and using different methods to enter variables into the model (data not shown). The type of antiseizure medication was not associated in this model. Given the association of cumulative postictal WHC and duration of sedation, we studied the cumulative doses of midazolam and propofol in patients who received both sedatives. Figure 2e, f illustrate the associations of propofol doses (mg/kg/h), cumulative postictal WHC grade, and midazolam doses (mg/kg/h) and suggests a higher risk of encephalopathy associated with higher midazolam but not with higher propofol doses. The average doses given were 0.11 mg/kg/h midazolam (IQR 0.02–0.19) and 2.2 mg/kg/h propofol (IQR 1.53–3.05 mg/kg/h) in patients with cumulative postictal WHC grade above median, and 0.03 mg/kg/h midazolam (IQR 0.01–0.06 mg/kg/h) and 2.62 mg/kg/h propofol (IQR: 1.69–3.28 mg/kg/h) in patients with cumulative postictal WHC below median (*p* = 0.007 for midazolam and *p* = 0.09 for propofol, Kruskal–Wallis test).

**Table 2:** Risk factors for prolonged postictal encephalopathy (*n* = 232)

Demographic		Count or mean	Percentage (%) or IQR	Count or mean	Percentage (%) or IQR	*p* value	Corrected *p* value^a^
		Cumulative WHC grade 21–69 (above median)		Cumulative WHC grade 0–20 (below median)			
Sex	Female	59	50.9	59	50.9	n.s	n.s
	Male	57	49.1	57	49.1		
Age	< 40	6	5.2	7	6.0	n.s	n.s
	40–70	54	46.6	43	37.1		
	> 70	56	48.3	66	56.9		
Etiology	Acute stroke	15	12.9	8	6.9	**0.001**	**0.04**
	Infectious	19	16.4	3	2.6		
	Intoxication/metabolic	19	16.4	19	16.4		
	Other acute symptomatic	8	6.9	3	2.6		
	Remote symptomatic	31	26.7	43	37.1		
	Progressive	12	10.3	23	19.8		
	Cryptogenic	12	10.3	10	8.6		
	Electroclin syndromes	0	0.0	7	6.0		
Worst seizure type	Focal (un)aware/absence	34	29.3	46	39.7	**< 0.001**	**0.03**
	Tonic–clonic	35	30.2	54	46.6		
	NCSE in coma	47	40.5	16	13.8		
Treatment	Intensive care unit	43	37.1	20	17.2	**< 0.001**	n.s
	Standard ward	73	62.9	96	82.8		
NCSE (Salzburg criteria)	Definite/possible NCSE	19	16.7	24	24.7	n.s	n.s
	No NCSE	95	83.3	73	75.2		
Patient not treated at ICU for SE (n = *169)*							
	Duration of SE (h)	113.0	36.8–153.6	94.6	6.5–99.0	0.002	n..s.^b^
Patient treated at ICU for SE (n = *63)*							
	Propofol treatment	27	84.4	16	84.2	n.s	–
	Midazolam treatment	25	78.1	15	78.9	n.s	–
	Duration of SE (h)	189.2	45.5–205.2	52.9	20.0–77.0	0.01	n.s
	Duration of sedation (h)	65.3	18.5–87.0	13.6	1.9–14.4	**< 0.001**	**0.02** ^b^
	Sedation after cessation of SE, median (h)	14.5	1.0–26.0	5.0	0.0–63.0	n.s	–
	Number of sedation cycles	2.2	1.0–3.0	0.9	1.0–1.0	**< 0.001**	–

Bold numbers indicate *p*-values <0.05

*ICU* intensive care unit, *IQR* interquartile range, *NCSE* nonconvulsive status epilepticus, *n.s.* not significant, *SE* status epilepticus, *WHC* West Haven criteria

^a^Logistic regression model correcting for the parameters in the table with *p* value

^b^Values for all patients were used in the model

**Table 3:** Clinical characteristics associated with the degree of postictal encephalopathy (linear regression model)

Characteristic	Corrected standardized coefficients	95.0% confidence interval for *B*		Significance	Variance inflation factor
	Beta	Lower bound	Upper bound		
Constant		− 11.850	17.029	0.724	
Etiology	− 0.157	− 2.275	− 0.295	**0.011**	1.056
Time with sedation of any kind (all pts.)	0.219	0.023	0.184	**0.012**	2.110
Propofol mg/kg/h	0.060	− 1.426	3.565	0.399	1.401
Midazolam mg/kg/h	0.075	− 23.656	80.400	0.284	1.371
Age (at admission)	0.180	0.070	0.408	**0.006**	1.167
SE duration (h)	0.002	− 0.018	0.018	0.984	1.617
Sex	0.008	− 4.602	5.247	0.897	1.094
CCI total	0.061	− 0.403	1.217	0.323	1.063
mRS before SE episode	0.141	0.280	3.482	**0.022**	1.035
Worst seizure type? (STESS)	0.178	1.339	7.751	**0.006**	1.133

Bold number indicate *p*-values <0.05

Model summary: R2: 0.21, *F* = 5.879

*CCI* Charlson comorbidity index (without age), *mRS* modified Rankin scale, *SE* status epilepticus, *STESS* status epilepticus severity score﻿

## Discussion

Here, we showed that encephalopathy after SE is a common and severe complication that is strongly associated with long-term mortality if unresolved at discharge. Three major factors contributed to the postictal encephalopathy in our cohort: etiology, (midazolam-) sedation in ICU, and premorbid condition (age, modified Rankin Scale before admission).

We found no consistent association with any of the seizure-related parameters investigated. Neither the type of SE (convulsive vs. NCSE), duration of SE, nor ictal MR changes showed a significant independent association with the postictal cumulative postictal WHC. Surprisingly, the association of duration of SE and encephalopathy was weak (Table 2) and did not reach statistical significance in the regression models applied. This supports the concept that the postictal encephalopathy is mainly the result of premorbid condition, etiology, and treatment in the ICU. The main problem challenging the interpretation of our results, however, is the complex interaction between the factors and that our study does not allow sound pathophysiological conclusions or establishing cutoffs suitable for clinical use. Factors indicative of more severe SE were associated with a higher cumulative postictal WHC (Table 2), but they were also associated with each other. Mathematical models such as the linear regression model used in this study may allow correcting for some of the interactions, but their outcome substantially depend on the parameters introduced.

Therefore, it is important that our results are in line with a previously published studies, e.g., by Baumann et al. [15], who found a significant association of ICU treatment and benzodiazepine doses and the odds for postictal delirium. Like in our cohort, the association of delirium and duration of SE did not reach statistical significance. Two other factors identified in our study, age and etiology, are well-known contributors to the postictal state, too, as summarized by Theodore [13].

**Fig. 3:**
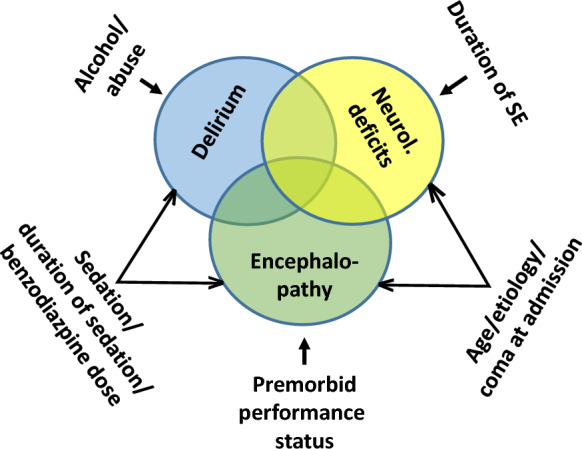
Simplified model illustrating the contributors to the different dimensions of the postictal state. SE, status epilepticus

Postictal encephalopathy is likely directly linked to the postictal state given that the postictal EEG often displays an encephalopathic pattern [10]. However, it is only one part of the spectrum of postictal symptoms [26] and is not the same as new neurological deficits. Further, transient mild to moderate postictal encephalopathy during the first 14 days does not strongly correlate with long-term survival (Supplementary Fig. 2b). Based on own clinical experience, the partially overlapping scores for delirium [15], new neurological deficits [9], and postictal encephalopathy likely cover most of this spectrum that may differ substantially between patients. Risk factors differ for the three dimensions of the spectrum. For postictal delirium, Baumann et al. [15] identified alcohol/drug dependence, high benzodiazepine doses, induced coma, and days of intubation as independent risk factors for postictal delirium. Roberg et al. [9] showed that etiology, age, coma at onset, duration of SE were critical for the development of new neurological deficits after SE. In this work, etiology, coma at arrival, duration of sedation, age, and premorbid disability were identified as independent risk factors for postictal encephalopathy. The overlap but also the differences between the identified risk factors for the different postictal phenomena are summarized in Fig. 3 providing a simplified overview of the major contributor to the postictal state.

It is difficult to speculate on the mechanisms contributing to the postictal encephalopathy. Clearly, etiology appears to contribute and remains an independent risk factor in a linear regression model. Especially inflammatory and infectious etiologies were associated with severe/prolong encephalopathy. Larger cohorts are, however, necessary, to account for the severity of the underlying disease. Indirect effects, e.g., the use of high-dose midazolam due aggressive underlying etiologies, complication of intensive care (e.g., pneumonia, antibiotics), and the need for more aggressive treatment due to diagnostic delays may have influenced the results, too.

The retrospective assessment of clinical parameters and the key outcome measure¸ WHC grade, is a limitation of our study. We used different approaches to reduce the impact of the retrospective assessment. Using a single and de facto blinded rater for all assessment of WHC helped to avoid interrater bias, rerating of the first 15 patients made a learning bias unlikely and confirmed a high retest reliability. Different, independent rater assessed all other parameters allowing for unbiased analyses of the data. The second main challenge was the correct quantification and definition of the “postictal encephalopathy” given the lack of established scores for encephalopathy after SE. We chose to use the well-established WHC [21], which previously allowed the successful identification of patients with hyperammonemia after high-dose valproic acid treatment in patients with SE but does not sufficiently cover different aspects of delirium [20]. Although this score was developed for hepatic encephalopathy, it appeared to be feasible for a retrospective analyses of the patient’s medical records due to its few but well-defined criteria. To estimate the extent of this dynamic state, we chose the cumulative postictal WHC grade during the first 14 days after SE. Given that a substantial proportion of patients were discharged or died before postictal day 14, several assumptions were necessary. We chose to give a WHC grade of zero for all patients after discharge being fully aware that we likely underestimated the degree of encephalopathy in this group. Conversely, deceased patients received a fictive score of 5. Using this approach, the cumulative WHC grade reliably distinguished patients with prolonged and severe postictal encephalopathy from patients with no or mild encephalopathy. Although EEG (20–30 min) was routinely performed after SE in patients with impaired consciousness to exclude NCSE [28], intermitted seizures not detected by spot EEG are difficult to exclude and represent an additional limitation. Further, we did not assess the EEGs after SE systematically with respect to the newly defined concept of an ictal-interictal continuum [27]. It is likely that a subgroup of patients with symptoms of encephalopathy had EEG patterns within this continuum and it is tempting to speculate that these patients had a worse prognosis. We tried to address this limitation by excluding patients with uncertain end of SE (Supplementary Fig. 1).

Despite these limitations, our results were consistent and plausible, and may contribute to the ongoing discussion on the optimal treatment of patients with SE [29–31]. Pending reproduction in independent cohorts, we think that our study has two major clinical implications. (1) Prolonged postictal encephalopathy is mainly due to etiology, duration of sedation, and the premorbid condition and age. Thus, rapid improvement is unlikely in predisposed patients, and these patients will need time to recover. (2) Sedation with high doses of midazolam may at worst increase the risk of a prolonged postictal encephalopathy. If higher doses are required to achieve seizure suppression, we therefore think that addition of other agents like ketamine—due to its lesser sedative effects and the increasing body of supportive evidence indicating its effectiveness in terminating seizures [32–34]—might be considered to avoid long-lasting encephalopathies in elderly patients with premorbid disabilities.

In conclusion, postictal encephalopathy after SE is common and mostly transient. Prolonged postictal encephalopathy is associated with poorer long-term survival. The underlying etiology, long sedation, high age, and poor premorbid condition are major risk factors for its development.

### Supplementary Information

Below is the link to the electronic supplementary material.

Supplementary Fig. 1: STROBE compliant flow chart for patient screening, identification, and selection. SE = status epilepticus, EEG = electroencephalogram, ICU = intensive care unit, WHC = West Haven Criteria. (PPTX 113 KB)

Supplementary file2 (DOCX 16 KB)
