# Supplementary material for: Postictal Encephalopathy After Status Epilepticus: Outcome and Risk Factors
Source: Neurocrit Care. 2023 Nov 8;40(3):1025–35. doi: 10.1007/s12028-023-01868-1 (PMC11147838; doi:10.1007/s12028-023-01868-1)
Supplement: Supplementary file 1 — Supplementary Fig. 1: STROBE compliant flow chart for patient screening, identification, and selection. SE = status epilepticus, EEG = electroencephalogram, ICU = intensive care unit, WHC = West Haven Criteria. (PPTX 113 KB) [file 12028_2023_1868_MOESM1_ESM.pptx]

## Slide 1
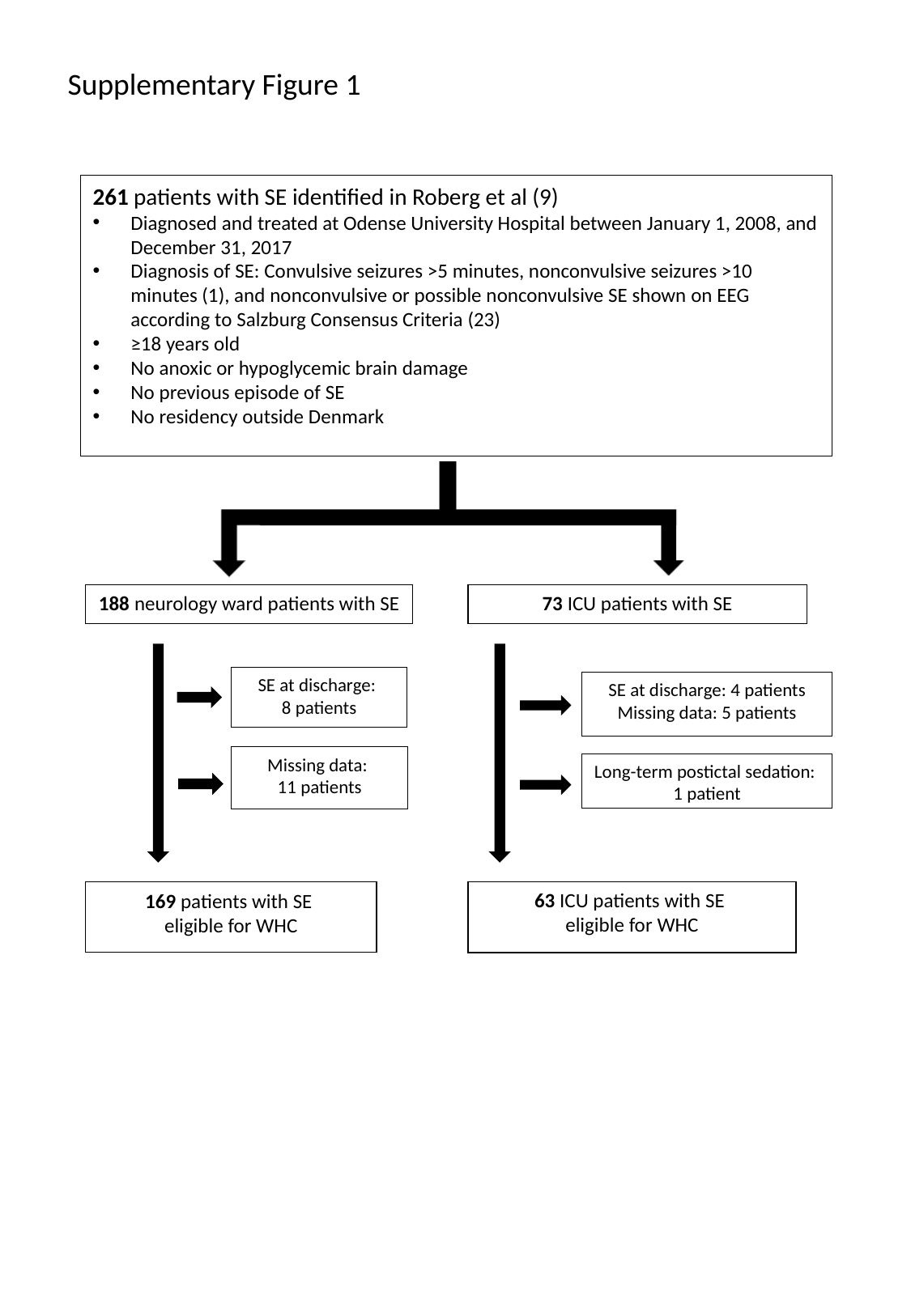

Supplementary Figure 1
261 patients with SE identified in Roberg et al (9)
Diagnosed and treated at Odense University Hospital between January 1, 2008, and December 31, 2017
Diagnosis of SE: Convulsive seizures >5 minutes, nonconvulsive seizures >10 minutes (1), and nonconvulsive or possible nonconvulsive SE shown on EEG according to Salzburg Consensus Criteria (23)
≥18 years old
No anoxic or hypoglycemic brain damage
No previous episode of SE
No residency outside Denmark
73 ICU patients with SE
188 neurology ward patients with SE
SE at discharge:
8 patients
SE at discharge: 4 patients
Missing data: 5 patients
Missing data:
11 patients
Long-term postictal sedation:
1 patient
63 ICU patients with SE
eligible for WHC
169 patients with SE
eligible for WHC

## Slide 2
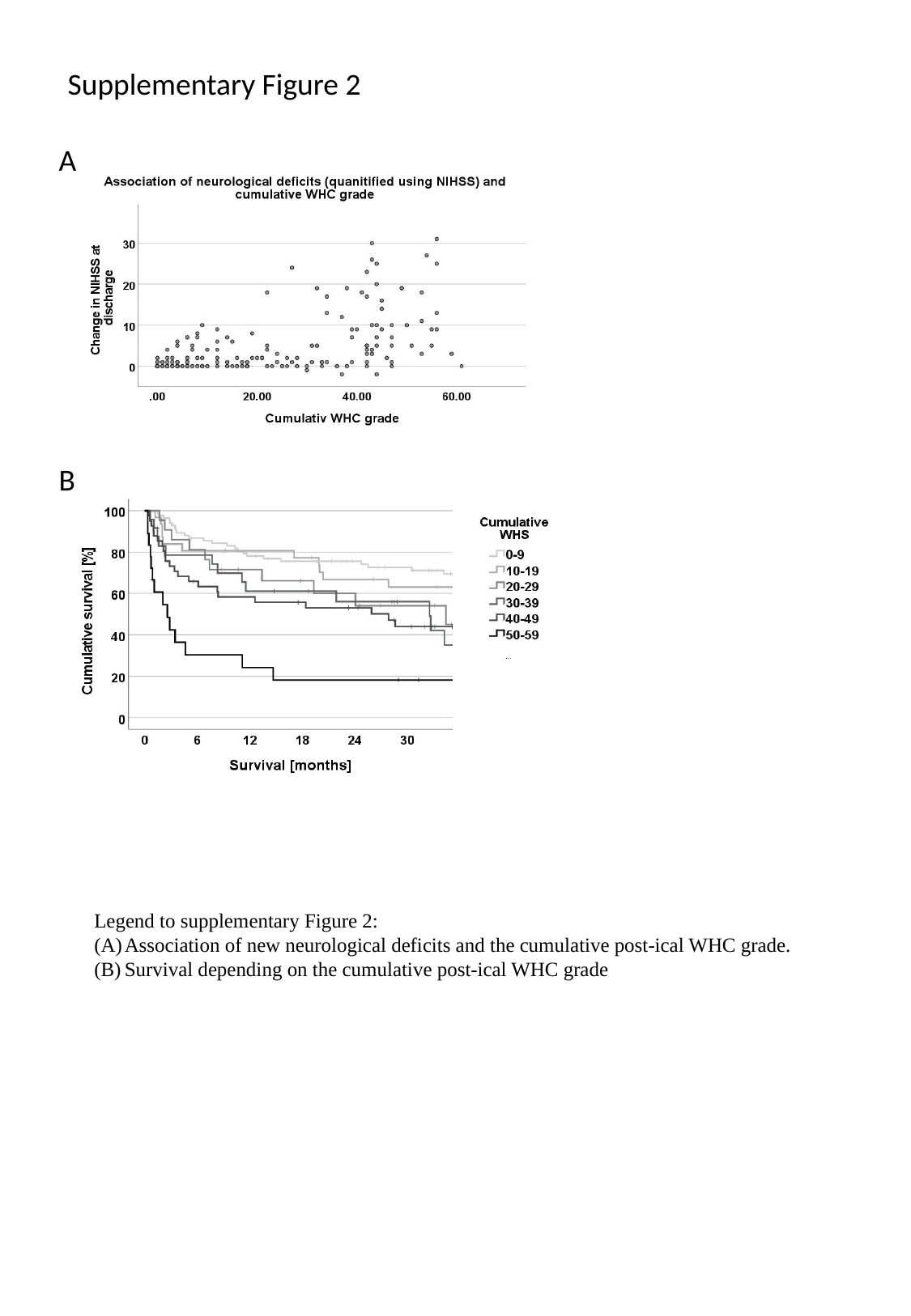

Supplementary Figure 2
A
B
Legend to supplementary Figure 2:
Association of new neurological deficits and the cumulative post-ical WHC grade.
Survival depending on the cumulative post-ical WHC grade
