# Supplementary material for: Postictal Encephalopathy After Status Epilepticus: Outcome and Risk Factors
Source: Neurocrit Care. 2023 Nov 8;40(3):1025–35. doi: 10.1007/s12028-023-01868-1 (PMC11147838; doi:10.1007/s12028-023-01868-1)
Supplement: Supplementary file 2 — Supplementary file2 (DOCX 16 KB) [file 12028_2023_1868_MOESM2_ESM.docx]

**Supplementary Table 1:** Etiology of status epilepticus in included patients

| Etiology | Count | N % |
| --- | --- | --- |
| Acute stroke | 23 | 10.3% |
| Meningitis and infectious encephalitis  -viral  -bacterial  -unknown/other | 22  4  13  5 | 9.8% |
| Intoxication and metabolic | 37 | 16.5% |
| Other acute symptomatic  -traumatic brain injury | 11  8 | 4.9%  3.6% |
| Post-stroke epilepsy | 61 | 27.2% |
| Other remote symptomatic | 13 | 5.8% |
| Malignant brain tumors | 30 | 13.4% |
| Other progressive causes (including dementia) | 5 | 2.2% |
| Cryptogenic | 22 | 9.8% |

**Supplementary Table 2:** Risk factors for prolonged post-ictal encephalopathy (n=232)

|  | | Median cumulative West Haven Criteria (Interquartile range) | | p-value |
| --- | --- | --- | --- | --- |
| Sex | Female | 20.5 | (6.0-42.0) | n.s.* |
|  | Male | 20.0 | (6.0-44.0) |  |
| Age | <40 | 12.0 | (6.0-43.0) | **0.04^#^** |
|  | 40-70 | 25.0 | (9.0-43.0) |  |
|  | >70 | 16.0 | (4.0-42.0) |  |
| Etiology | Acute stroke | 31.0 | (9.0-43.0) | **<0.001^#^** |
|  | Infectious | 43.0 | (28.0-49.0) |  |
|  | Intoxication/metabolic | 21.5 | (7.0-42.0) |  |
|  | Other acute symptomatic | 43.0 | (7.0-56.0) |  |
|  | Remote symptomatic | 16.5 | (6.0-34.0) |  |
|  | Progressive | 8.0 | (2.0-36.0) |  |
|  | Cryptogenic | 35.0 | (6.0-49.0) |  |
|  | Electroclin. syndromes | 2.0 | (0.0-6.0) |  |
| Worst seizure type | focal (un)aware/absence | 15.5 | (4.0-35.5) | **<0.001^#^** |
|  | tonic-clonic | 14.0 | (4.0-38.0) |  |
|  | NCSE in coma | 42.0 | (17.0-50.0) |  |
| Treatment | Intensive care unit | 43.0 | (15.0-47.0) | **<0.001*** |
|  | Standard ward | 15.0 | (5.0-37.0) |  |
| NCSE (Salzburg criteria) | Definite/possible NCSE | 18.0 | (6.0-44.0) | n.s.* |
|  | No NCSE | 27.5 | (7.5-43.0) |  |

*Mann-Whitney U test; #Kruskal Wallis test
